# The Major Ciliary Isoforms of RPGR Build Different Interaction Complexes with INPP5E and RPGRIP1L

**DOI:** 10.3390/ijms22073583

**Published:** 2021-03-30

**Authors:** Christine Vössing, Paul Atigbire, Jannis Eilers, Fenja Markus, Knut Stieger, Fei Song, John Neidhardt

**Affiliations:** 1 Human Genetics, Faculty VI-School of Medicine and Health Sciences, University of Oldenburg, 26129 Oldenburg, Germany; christine.voessing@uni-oldenburg.de (C.V.); paul.atigbire@uol.de (P.A.); jannis.eilers@uni-oldenburg.de (J.E.); fei.song@uol.de (F.S.); 2 Junior Research Group, Genetics of Childhood Brain Malformations, Faculty VI-School of Medicine and Health Sciences, University of Oldenburg, 26129 Oldenburg, Germany; fenja.markus@uni-oldenburg.de; 3 Department of Ophthalmology, Justus-Liebig-University Giessen, 35390 Giessen, Germany; Knut.Stieger@uniklinikum-giessen.de; 4 Research Center Neurosensory Science, University of Oldenburg, 26129 Oldenburg, Germany; 5 Joint Research Training Group of the Faculty of Medicine and Health Sciences, University of Oldenburg, Germany and the University Medical Center Groningen, 9700 Groningen, The Netherlands

**Keywords:** RPGR, cilium, ciliary network, retinitis pigmentosa, isoform, splicing, RPGRIP1L, INPP5E, PDE6D, interaction complex

## Abstract

X-linked retinitis pigmentosa (XLRP) is frequently caused by mutations in the *retinitis pigmentosa GTPase regulator* (*RPGR*) gene. A complex splicing process acts on the *RPGR* gene resulting in three major isoforms: *RPGR^ex1-19^*, *RPGR^ORF15^* and *RPGR^skip14/15^*. We characterized the widely expressed, alternatively spliced transcript *RPGR^skip14/15^* lacking exons 14 and 15. Using the CRISPR/eSpCas9 system, we generated HEK293T cell lines exclusively expressing the *RPGR^skip14/15^* transcript from the endogenous *RPGR* gene. *RPGR^ex1-19^* and *RPGR^ORF15^* were knocked out. Immunocytochemistry demonstrated that the RPGR^skip14/15^ protein localizes along primary cilia, resembling the expression pattern of RPGR^ex1-19^. The number of cilia-carrying cells was not affected by the absence of the RPGR^ex1-19^ and RPGR^ORF15^ isoforms. Co-immunoprecipitation assays demonstrated that both RPGR^ex1-19^ and RPGR^skip14/15^ interact with PDE6D, further supporting that RPGR^skip14/15^ is associated with the protein networks along the primary cilium. Interestingly, interaction complexes with INPP5E or RPGRIP1L were only detectable with isoform RPGR^ex1-19^, but not with RPGR^skip14/15^, demonstrating distinct functional properties of the major RPGR isoforms in spite of their similar subcellular localization. Our findings lead to the conclusion that protein binding sites within RPGR are mediated through alternative splicing. A tissue-specific expression ratio between RPGR^skip14/15^ and RPGR^ex1-19^ seems required to regulate the ciliary concentration of RPGR interaction partners.

## 1. Introduction

Retinitis Pigmentosa (RP) is an inherited retinal disorder, characterized by the progressive loss of rod and cone photoreceptor cells, frequently leading to legal blindness. RP can either occur as an isolated non-syndromic disease, termed simplex RP, or may be part of a complex syndromic disease, e.g., Usher syndrome, Joubert syndrome, Bardet-Biedl syndrome or primary ciliary dyskinesia [1,2,3,4]. The clinical phenotype of RP can be caused by mutations in more than 60 genes (see Retinal Information Network, Retnet, https://sph.uth.edu/retnet, accessed on 15 January 2021). Inheritance patterns may follow autosomal-dominant, autosomal-recessive or X-linked traits. X-linked RP (XLRP) is among the most severe forms of RP, frequently associated with early disease onset and rapid progression [5]. Up to 80% of all XLRP cases and approximately 10–20% of familial RP cases are associated with mutations in the *retinitis pigmentosa GTPase regulator* gene (*RPGR*; OMIM #312610) [6,7].

The human *RPGR* gene consists of at least 22 exons encoding three major transcript isoforms: *RPGR^ex1-19^*, *RPGR^skip14/15^*, and *RPGR^ORF15^*. *RPGR^ex1-19^* is the widely expressed full-length isoform of *RPGR* which includes all exons from 1 through 19, whereas *RPGR^skip14/15^* is generated by the alternative splicing of exons 14 and 15. Notably, skipping of exons 14 and 15 during splicing leads to an in-frame deletion in *RPGR* transcripts. The alternative splicing of *RPGR* further generates several retina-enriched transcripts, one of which is *RPGR^ORF15^*. In *RPGR^ORF15^*, exon 15 is extended into intron 15 to generate the 3′-end exon ′open reading frame 15’ (ORF15), an exon that has been found to be a hotspot for RP-associated mutations [7,8,9,10,11]. Additionally, *RPGR* exon 15a-containing transcripts were mostly detected in the human retina [8], whereas exon 9a expression was found to be enriched in cone photoreceptors [9].

The constitutively expressed *RPGR^ex1-19^* transcript encodes a widely expressed 815 amino acid protein. The protein shows a characteristic dot-like expression pattern along the transition zone and axoneme of primary cilia [12]. Similarly, the retina-specific *RPGR^ORF15^* isoform was found to localize predominantly along the connecting cilium (CC) of photoreceptor cells [13,14]. RPGR^ex1-19^ contains an isoprenylation motif (CAAX) at the C-terminal cysteine 812, a lipid-based modification involved in the regulation of RPGR’s cellular localization and its association to interaction complexes [15]. All RPGR isoforms share the same N-terminal region including a tandem repeat structure that is homologous to the regulator of chromosome condensation 1 (RCC1) motif, termed RCC1-like domain (RLD). It is speculated that the RLD, encoded by exons 2 to 11, might function as a guanine nucleotide exchange factor (GEF) for small GTPases, but so far, no GTPase activity has been associated with RPGR [6,16].

Several RPGR interaction partners have been identified. It was shown that the proteins SMC1 and SMC3 (structural maintenance of chromosomes protein 1 and 3) interact with the RCC1-like domain of RPGR [17]. Furthermore, it was documented that RPGRIP1 (retinitis pigmentosa GTPase regulator-interacting protein 1) interacts with RPGR^ORF15^, a complex that is important for its localization to the photoreceptor CC [18,19]. RPGRIP1L (RPGR-interacting protein 1-like), PDE6D (phosphodiesterase 6D) and INPP5E (inositol polyphosphate-5-phosphatase E) were reported to interact with two RPGR isoforms: RPGR^ex1-19^ and RPGR^ORF15^ [15,20]. An important interaction partner that influences the ciliary localization of RPGR^ex1-19^ is PDE6D [15,20]. Initially, it was described that the RCC1-like domain interacts with PDE6D [21,22]. More recently, studies suggested that PDE6D binds to the prenylated C-terminal part of RPGR^ex1-19^ and that INPP5E associates with the N-terminal region of RPGR [20].

The third major isoform is represented by RPGR^skip14/15^, an isoform that results from the skipping of exons 14 and 15 during splicing. This isoform is widely expressed in several human tissues [8,11], a distribution that is confirmed by publicly accessible RNAseq data sets (GTExPortal, GTEx Transcript browser, RPGR splice junction 6, https://www.gtexportal.org/home/transcriptPage, accessed on 15 January 2021). Although *RPGR^skip14/15^* seems to be expressed at levels comparable to the other two constitutive *RPGR* isoforms, the relative abundance varies between tissues [11]. Surprisingly, little is known about this alternatively spliced *RPGR^skip14/15^* transcript and its protein. Therefore, we have undertaken further studies to compare the functional properties of two major RPGR isoforms: RPGR^ex1-19^ and RPGR^skip14/15^. The CRISPR/eSpCas9 genome editing system was used to generate HEK293T cell lines exclusively expressing the alternatively spliced *RPGR^skip14/15^* transcript. Our data show that RPGR^skip14/15^ locates to primary cilia and suggests selective interaction with RPGR-binding partners.

## 2. Results

### 2.1. The RPGR^skip14/15^ Transcript Is Widely Expressed in Several Human Tissues

**Figure 1:**
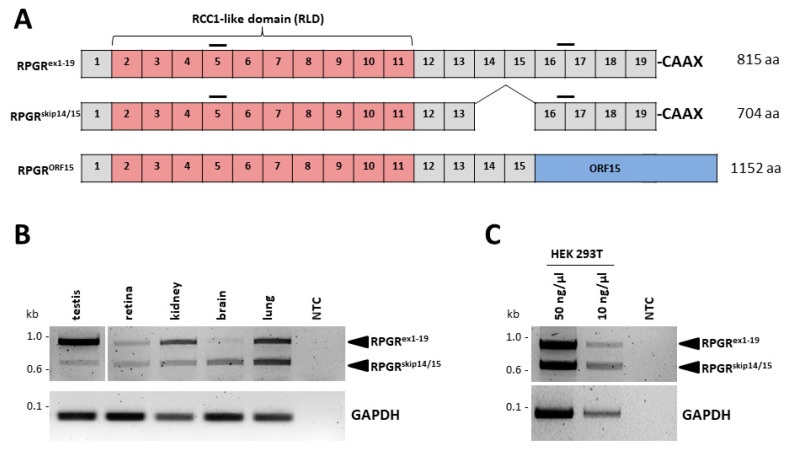
Analysis of retinitis pigmentosa GTPase regulator (*RPGR*) isoforms. (**A**) Schematic representations of the three major *RPGR* isoforms expressed in humans: *RPGR^ex1-19^*, *RPGR^skip14/15^* and *RPGR^ORF15^*. Exons are represented by boxes. The 5′ part of the transcript isoforms contain the RCC1-like domain encoded by exons 2 to 11 (marked in red). The 5′ end of exon 19 in *RPGR^ex1-19^* and *RPGR^skip14/15^* encodes an isoprenylation motif (CAAX). Exon ORF15 of *RPGR* is shown in blue. Horizontal bars represent primer locations used in RT-PCR analyses. (**B**) RT-PCR analysis was performed to detect *RPGR^ex1-19^* and *RPGR^skip14/15^* from cDNA of several human tissues. Both *RPGR^ex1-19^* and *RPGR^skip14/15^* are widely expressed and show varying relative expression levels. The PCR-product of 958 bp corresponds to the isoform *RPGR^ex1-19^*. The 625 bp product represents *RPGR^skip14/15^*. (**C**) RT-PCR on HEK293T cells (using 50 ng/µL or 10 ng/µL cDNA) suggested approximately equal expression levels of *RPGR^ex1-19^* and *RPGR^skip14/15^*. NTC: non-template control. Glyceraldehyde 3-phosphate dehydrogenase (*GAPDH*): loading control.

The *RPGR^skip14/15^* transcript encodes a 704 amino acid protein. The transcript comprises exons 1 to 13 and exons 16 to 19, but lacks/skips exons 14 and 15, which leads to an in-frame deletion of corresponding protein-coding regions (Figure 1A). The protein isoform (RPGR^skip14/15^) contains the N-terminal RCC1-like domain, which is also present in the isoforms RPGR^ex1-19^ and RPGR^ORF15^. C-terminally, RPGR^skip14/15^ as well as RPGR^ex1-19^ contain the exon 19-encoded isoprenylation motif (CAAX) at cysteine 812. To understand the differences caused by the alternative splicing of exons 14 and 15 in *RPGR*, we compared RPGR^skip14/15^ and RPGR^ex1-19^ with a focus on primary cilium-related properties.

We verified the expression of the *RPGR^skip14/15^* isoform in a selection of human tissues. To estimate the expression ratio between the *RPGR^ex1-19^* and *RPGR^skip14/^*^15^ isoforms in different tissues, we performed RT-PCRs that amplified both transcripts in the same reaction (Figure 1B). A PCR product of 958 bp was detected for the *RPGR^ex1-19^* isoform, while *RPGR^skip14/15^* generated a PCR product of 625 bp. In the human retina, the intensity ratio between PCR products of both *RPGR* isoforms suggested similar expression levels. In contrast, the human testis, kidney and brain showed unequal expression ratios; *RPGR^skip14/15^* expression was lower in the testis and kidney, but comparably higher in the brain (Figure 1B, Figure S2). RNA sequencing data published in the GTEx Transcript Browser (https://www.gtexportal.org/home/transcriptPage, accessed on 15 January 2021) are in line with these estimates. The read counts of exon junction 13 to 16 and 15 to 16 showed ratios of approximately 1 to 50 in testis, 1 to 3 in kidney, 1 to 10 in lung, and between 1 to 10 and 1 to 1 in different brain regions. Together, these data suggest that the *RPGR^skip14/15^* isoform is a widely expressed transcript with variable expression levels among different human tissues. Additionally, HEK293T cells presented with similar expression levels of *RPGR^skip14/15^* and *RPGR^ex1-19^* (Figure 1C). Of note, it can neither be excluded that the RT-PCR preferentially amplified the shorter transcript, nor that the tissue resources and preparations influenced the expression ratios between *RPGR^skip14/15^* and *RPGR^ex1-19^*. Sanger sequence analysis confirmed the identity of the two isoforms from all tissues/cell lines (Figures S3 and S4).

### 2.2. Generation of an Isogenic Cell Line Exclusively Expressing the RPGR^skip14/15^ Transcript

**Figure 2:**
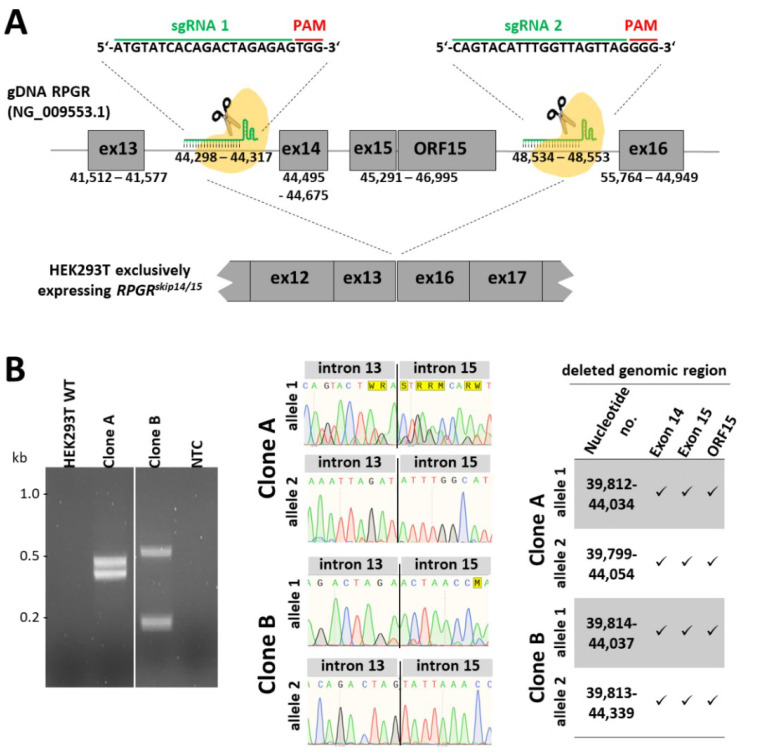

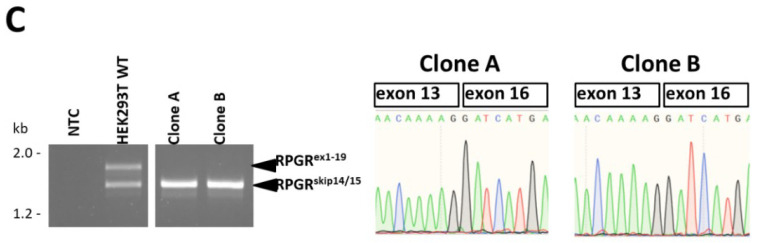
Generation of an isogenic HEK293T cell line exclusively expressing the *RPGR^skip14/15^* transcript. (**A**) Schematic illustration showing the genomic region of the *RPGR* gene including exons 13 through 16. The first sgRNA (sgRNA1) is located in intron 13 close to exon 14, the second sgRNA (sgRNA2) binds downstream of the ORF15 region in intron 15. The cleavage of both sgRNAs induces a deletion of genomic regions including exon 14, 15 and ORF15 from the HEK293T genome. The deletion is expected to result in the exclusive expression of the *RPGR^skip14/15^* transcript. (**B**) PCR fragments showing the genomic alterations caused by the CRISPR/eSpCas9 system. Clone A and clone B contain genomic deletions in which exons 14, 15 and ORF15 were removed from the *RPGR* gene. Electropherograms document the break points of the deletions in intron 13 and 15 on the X-chromosomes. Of note, the mutated alleles in clone A seem to be concomitantly purified from the agarose gel, which might have caused the overlapping sequencing profiles in allele 1 of clone A. The nucleotide positions of the deletion break points are shown in the table on the right. RPGR reference sequence: NG_009553.1. (**C**) RT-PCR analyses of *RPGR* expression in clones A and B. The RT-PCR product of 1772 bp corresponds to *RPGR^ex1-19^*, which is only detectable in wild-type HEK293T cells. The shorter 1439 bp RT-PCR product represents *RPGR^skip14/15^*. Clone A and clone B exclusively expressed the *RPGR^skip14/15^* transcript, while untreated HEK293T cells expressed both *RPGR* transcripts: *RPGR^ex1-19^* and *RPGR^skip14/15^*. Sanger sequencing reactions confirmed that exon 13 was spliced to exon 16. NTC = non-template control.

With the aim to engineer cellular clones that selectively express the *RPGR^skip14/^*^15^ isoform, we applied the CRISPR/eSpCas9 genome editing system and deleted the genomic region containing *RPGR* exons 14, 15 and ORF15 from X-chromosomal alleles in HEK293T cells. We designed two single-guide RNAs (sgRNAs) targeting the human *RPGR* locus at intron 13 and intron 15 (Figure 2A). Both sgRNAs-containing plasmids were equimolarly mixed and transfected into HEK293T cells. Single cell clones were isolated by fluorescence-activated cell sorting (FACS). The induced double strand breaks in *RPGR* (reference sequence: NG_009553.1) caused different deletions in clones A and B (Figure 2B). The deletions are as follows: (i) deletion 1 in clone A: 4222 bp deletion (39,812–44,034) and deletion 2 in clone A: 4255 bp deletion (39,799–44,054); (ii) deletion 1 in clone B: 4223 bp deletion (39,814–44,037) and deletion 2 in clone B: 4526 bp deletion (39,813–44,339). In summary, we generated two independent HEK293T cell lines, named clone A and clone B, both of which lack parts of the genomic region between *RPGR* exon 13 and exon 16.

The selected sgRNAs were designed to specifically bind the *RPGR* target sites and to simultaneously avoid additional binding sites in the human genome. We verified whether potential off-target sites were altered by the CRISPR/eSpCas9 approach. The next best binding was bioinformatically predicted with 2 to 4 mismatches in the sgRNAs (Figure S1). These potential off-target sites, including several hundred base pairs of flanking genomic DNA, were sequenced. We did not detect sequence changes in 14 potential off-target sites (Figure S1).

### 2.3. CRISPR/eSpCas9-Mediated Clones A and B Exclusively Express the RPGR^skip14/15^ Isoform

Clones A and B were analyzed by RT-PCR and compared to untreated HEK293T control cells. We applied a primer combination that amplified both *RPGR* transcripts, *RPGR^ex1-19^* and *RPGR^skip14/15^*, in a single RT-PCR reaction (Figure 2C). The two *RPGR* isoforms were amplified with almost equal intensities from the cDNA of untreated HEK293T cells. In contrast, clone A and clone B selectively expressed the *RPGR^skip14/15^* transcript, whereas *RPGR^ex1-19^* was undetectable. Sanger sequencing verified the identity of the RT-PCR products and confirmed that *RPGR* exon 13 was directly spliced to exon 16 (Figure 2C).

**Figure 3:**
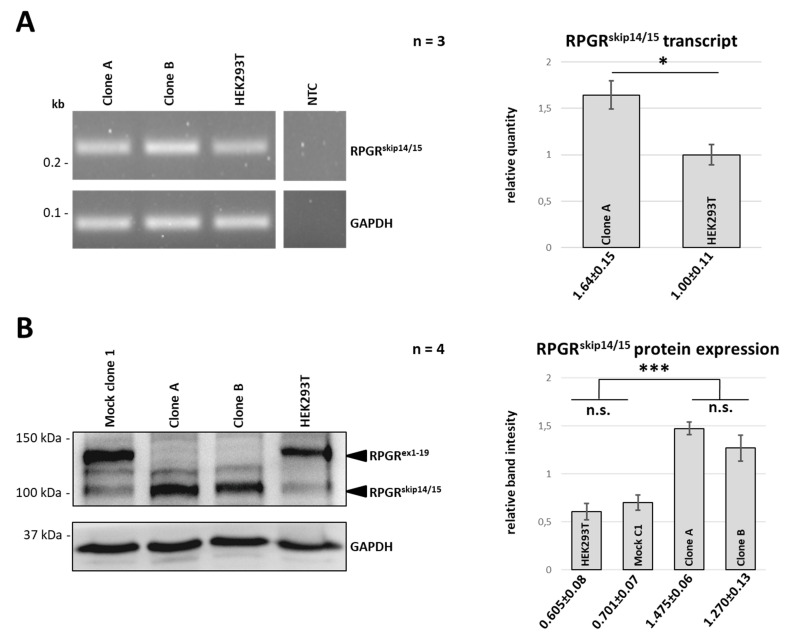
Expression analyses of RPGR^skip14/15^ in clone A and clone B. (**A**) RT-PCR analysis was performed with a primer combination that selectively amplified the *RPGR* transcript skipping exons 14 and 15 (*RPGR^skip14/15^*). A PCR-product of 214 bp corresponds to *RPGR^skip14/15^*. Approximately 1.6 times increased transcript levels of *RPGR^skip14/15^* were detected by quantitative RT-PCR analyses comparing clone A with wild-type HEK293T (n = 3). (**B**) Western blot analyses were performed with an RPGR-specific antibody that detected both RPGR protein isoforms: RPGR^ex1-19^ and RPGR^skip14/15^. The arrow heads indicate either the full-length RPGR^ex1-19^ protein with a size of approx. 135 kDa or the shorter RPGR protein RPGR^skip14/15^ with a size of around 115 kDa. In comparison to controls, quantitative assessment of the band intensities confirmed that the CRISPR/eSpCas9-mediated clones A and B show approximately 2 times higher protein levels of RPGR^skip14/15^. Western blot experiments were repeated four times (n = 4). Error bars represent standard deviation. n.s: non-significant, *: *p* ≤ 0.05, ***: *p* ≤ 0.001.

We further verified whether the genomic deletion of *RPGR* exons 14, 15 and ORF15 influenced the mRNA expression level of *RPGR^skip14/15^* by RT-PCR analyses. We established a primer combination which selectively amplifies *RPGR^skip14/15^* with a product size of 214 bp (Figure 3A). *RPGR^skip14/15^* transcript levels in clones A and B were compared to untreated HEK293T cells. Conventional RT-PCR analyses suggested increased levels of *RPGR^skip14/15^* in clone A and B. Quantitative RT-PCR showed that the CRISPR-mediated genomic deletion of exons 14, 15 and ORF15 in clone A resulted in approximately 1.6 times higher expression of *RPGR^skip14/15^*.

### 2.4. RPGR^ex1-19^ Protein Is Not Expressed in Clones A and B

Western blot analyses were performed to verify the RPGR protein expression in clones A and B (Figure 3B). We used a polyclonal antibody directed against epitopes located between E379 to N509 of the RPGR amino acid sequence (https://www.uniprot.org/uniprot/Q92834, accessed on 15 January 2021), a stretch of 131 amino acids encoded by exons 10 to 13. In wild-type HEK293T cells, the antibody detected RPGR^skip14/15^ and RPGR^ex1-19^ at approximately 115 and 135 kDa, respectively. In contrast, the CRISPR/eSpCas9-modified clones A and B only presented with RPGR^skip14/15^ signals, but lacked any detectable RPGR^ex1-19^ (Figure 3B). We quantitively compared the band intensities and found that the genomic modification of clones A and B caused an approximately 2 times increased expression of RPGR^skip14/15^ (Figure 3B). The band detected between RPGR^skip14/15^ and RPGR^ex1-19^ likely resulted from unspecific (non-RPGR) binding of the polyclonal antibody, as it was reproducibly detectable in every sample after longer exposure times (data not shown).

### 2.5. RPGR^skip14/15^ Locates along the Axoneme of a Primary Cilium

**Figure 4:**
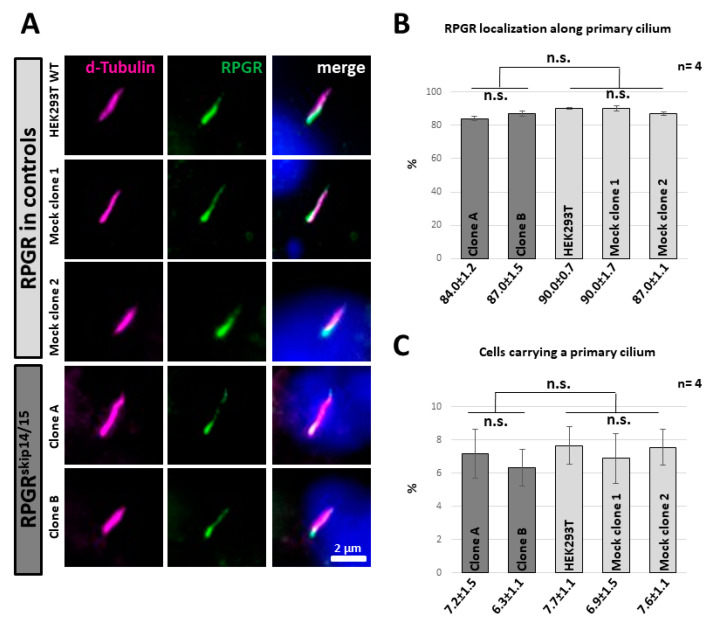
Ciliary localization of RPGR in clones A and B. (**A**) Immunocytochemistry staining of the primary cilium (d-tubulin, magenta) and subcellular localization of RPGR (green). Three independent control cell lines with endogenously expressed RPGR isoforms located RPGR along the axoneme of the cilium. Comparably, the CRISPR/eSpCas9-modified clones A and B, which selectively expressed RPGR^skip14/15^ from the endogenous *RPGR* gene, also showed RPGR signals along the axoneme. Cell nuclei were stained with DAPI (blue), included in the mounting media. (**B**) Quantitative assessment of cells that showed RPGR signals along the ciliary axoneme. Values between clones A and B and controls were not different (in percent). (**C**) Quantification of the number of cilia-carrying cells (in percent) indicated that the generation of primary cilia was not altered in clones A and B. Error bars represent standard deviation. Experiments were repeated 4 times (n = 4). n.s.: non-significant. Scale bar: 2 micrometers (µm).

We analysed the subcellular localization of RPGR^skip14/15^ at the primary cilium by immunocytochemistry. We detected RPGR^skip14/15^ along the axoneme of the primary cilium, partially co-localizing with the axonemal signals (Figure 4A, Figure S5), an observation that was consistent with previous studies in which we found RPGR in a dot-like expression pattern along the cilium in human dermal fibroblasts [12]. The strongest RPGR signals were found near the presumed transition zone of the primary cilium with the intensity of RPGR expression decreasing along the axoneme from ciliary base to ciliary tip. Significantly, both CRISPR/eSpCas9-modified cell lines showed RPGR^skip14/15^ along the ciliary axoneme, a localization that was highly similar to controls. These findings showed for the first time that endogenously expressed RPGR^skip14/15^ locates to the ciliary axoneme. The expression pattern of RPGR^skip14/15^ along the ciliary axoneme was verified in four independent experiments, in total analyzing 200 cilia per cell line (Figure 4B). Approximately 80 to 90% of the cilia-carrying cells showed the characteristic dot-like expression pattern of RPGR along the ciliary axoneme.

We asked whether the exclusive expression of RPGR^skip14/15^ influenced ciliogenesis and compared the amount of cilia-carrying cell among CRISPR/eSpCas9-modified cells and controls (Figure 4C). To stimulate the generation of cilia, we cultivated all cell lines in nutrition-reduced culture medium under highly comparable conditions. Approximately 7% of cells showed a primary cilium. Quantification of four independent experiments indicated no significant down- or upregulation among the HEK293T-derived cell lines, suggesting that neither ciliogenesis nor stability of primary cilia were strongly influenced by the selective expression of RPGR^skip14/15^ from the endogenous *RPGR* gene.

### 2.6. RPGR Isoforms Build Distinct Interaction Complexes

**Figure 5:**
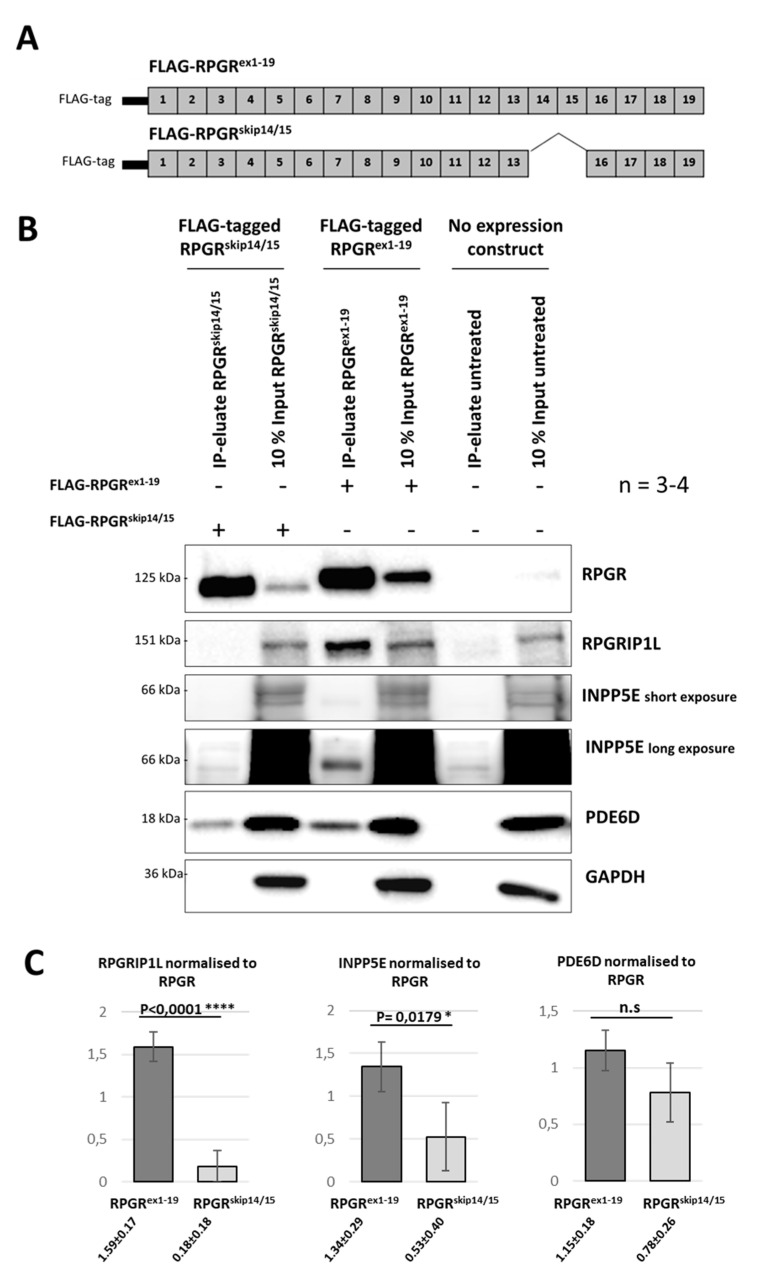
RPGR^skip14/15^ interacts with endogenous PDE6D, but not with INPP5E nor RPGRIP1L. (**A**) Schematic representation of RPGR expression constructs carrying N-terminal FLAG tags. FLAG-RPGR^ex1-19^ encodes the full-length RPGR, whereas FLAG-RPGR^skip14/15^ encodes the RPGR isoform skipping exon 14 and 15. (**B**) The FLAG-tagged RPGR constructs were transfected into HEK293T cells followed by co-immunoprecipitation (Co-IP) with magnetic FLAG beads. PDE6D, RPGRIP1L and INPP5E were detected in the Co-IP eluates and compared between the two RPGR isoforms (RPGR^skip14/15^ and RPGR^ex1-19^). RPGR^ex1-19^ associated with all three binding partners, whereas RPGR^skip14/15^ selectively binds to PDE6D. The analyses were repeated four to five times (n = 4–5). (**C**) Quantification of Co-IP signals. Signal intensities were normalized to RPGR. These quantitative results confirm that RPGR^skip14/15^ shows reduced binding affinity to RPGRIP1L and INPP5E. n.s: non-significant, *: *p* ≤ 0.05, ****: *p* ≤ 0.0001.

The ciliary localization of RPGR^skip14/15^ suggested that ciliary binding partners might associate with this isoform. It was previously described that RPGR^ex1-19^ interacts—among others—with the binding partners RPGRIP1L, INPP5E and PDE6D [10,15,20]. We performed co-immunoprecipitation (Co-IP) experiments to detect RPGR-binding partners using N-terminally FLAG-tagged expression constructs for both isoforms, RPGR^ex1-19^ and RPGR^skip14/15^ (Figure 5A). The pull-down of RPGR protein complexes (Figure 5B) was performed using magnetic anti-Flag beads. Co-IP-eluates showed enrichment of both FLAG-tagged RPGR^skip14/15^ and RPGR^ex1-19^ proteins. GAPDH served as a loading control. We confirmed that RPGR^ex1-19^ interacts with INPP5E, RPGRIP1L and PDE6D. In contrast, our results demonstrate that RPGR^skip14/15^ interacts with PDE6D, but not or significantly less with INPP5E and RPGRIP1L. This implies that the proteins RPGRIP1L and INPP5E were not part of interaction complexes of the RPGR isoform RPGR^skip14/15^.

## 3. Discussion

Alternative splicing is a cellular mechanism that allows for the expression of several different transcript isoforms from a single gene. A high number of mammalian genes undergo alternative splicing, thus contributing to protein diversity [23,24]. Alternative transcripts are frequently expressed in a cell-type-specific or time-dependent manner. They often mediate cell-type-specific functions, determine cellular localization or modulate the composition of interaction complexes [25,26].

A complex splicing pattern modifies the gene products of human *RPGR* resulting in heterogeneity among different alternative RPGR isoforms. In this study, we focused on the alternatively spliced isoform RPGR^skip14/15^, one of the major isoforms of *RPGR* expressed in several human tissues. Considering its widespread expression, surprisingly little is known about RPGR^skip14/15^. Using the CRISPR/eSpCas9 genome editing system, we generated HEK293T cell lines which exclusively express RPGR^skip14/15^. RPGR^skip14/15^ localized along the primary cilium and built complexes with PDE6D. In contrast to RPGR^ex1-19^, RPGR^skip14/15^ showed reduced affinity to INPP5E and RPGRIP1L. It seems likely that the in-frame deletion of exon 14 and 15 translates into an altered structure of the RPGR protein in which binding sites are either changed or not accessible for protein complexes containing partners such as INPP5E and RPGRIP1L.

As RPGR^skip14/15^ was able to enter the ciliary transition zone and was observed in the dot-like expression pattern along the axoneme, it seems likely that RPGR^skip14/15^ is part of ciliary cargo transported by the intraflagellar transport (IFT) pathway. Rao and colleagues identified a region of the human RPGR which is involved in its ciliary localization. It was shown that green fluorescence protein (GFP)-tagged constructs encoded by exon 16 to 19 of RPGR predominantly localized to primary cilia [20]. Further studies have suggested that GFP-tagged RPGR^ex1-19^ localizes to the primary cilium of RPE1 and IMCD3 cells, while expression constructs encoding exons 1 through 15 did not localize to cilia [15,20]. This study demonstrated that exons 14 and 15 are not essential for the ciliary localization of RPGR, an observation that was also suggested by GFP-tagged RPGR expression constructs encoded by exon 12 to 15 [20]. The ciliary localization of RPGR rather seems to be regulated by prenylation of cysteine 812 at the CAAX site encoded by exon 19. GFP-tagged expression constructs of RPGR^ex1-19^ harbouring a deletion of the CAAX motif did not localize to cilia [20]. These findings suggested that the ciliary localization of RPGR^skip14/15^, the isoform studied herein, is also regulated by the prenylation site encoded by exon 19 of *RPGR*. It has been described that the protein PDE6D regulates subcellular localization of several different prenylated ciliary proteins [27,28]. Mutations in the human *PDE6D* gene are associated with the ciliopathy Joubert syndrome-22, which is characterised by brain abnormalities and neurologic symptoms [29,30,31]. Furthermore, a PDE6D knockout cell line showed mis-localization of RPGR [15]; RPGR^ex1-19^ was unable to localize along the primary cilium anymore. These observations further support the hypothesis that the protein PDE6D binds to the prenylated RPGR^skip14/15^ and regulates its ciliary localization. We speculate that the interaction of PDE6D and RPGR^skip14/15^ is essential for the physiological function of both RPGR^skip14/15^ and the primary cilium. Our data suggest that a tissue-specific and well-balanced localization of RPGR-binding partners, e.g., like RPGRIP1L and INPP5E, is partially regulated by the expression level of RPGR^skip14/15^. Considering that many cellular signaling processes are influenced by the primary cilium, it seems likely that fine-tuning the isoform ratio and concentration of RPGR-binding partners at the cilium is of physiological relevance.

RPGR^skip14/15^ shares the N-terminal half (encoded by exons 1 to 13) with RPGR^ex1-19^ and RPGR^ORF15^ which also includes the entire RCC1-like domain. It has been described that the RCC1-like domain is important for the interaction between RPGR and several binding partners such as RPGRIP1L, PDE6D, and INPP5E [10,15,17,32]. Interestingly, we show herein that the RPGR^skip14/15^ isoform, with its RCC1-like domain, interacts with the protein PDE6D, but not or significantly less with the proteins RPGRIP1L and INPP5E.

PDE6D also binds to several other prenylated ciliary proteins including INPP5E. INPP5E is a phospholipid hydrolase that catalyzes the removal of phosphate groups from position 5 (of the inositol ring) in phophoinositides such as phosphatidylinositol (3,4,5)-trisphophate (PI-3,4,5-P_3_) and phosphatidylinositol (4,5)-bisphosphate (PI-4,5-P_2_) [33]. This modulation of the phosphoinositide content of the ciliary membrane by INPP5E influences ciliary functions including Hedgehog signalling [34,35,36]. Mutations in the INPP5E gene cause Joubert syndrome 1 characterized by mental retardation, retinal dystrophy and truncal obesity [37]. Interaction studies showed that INPP5E binds to RPGR^ex1-19^, to RPGR^ORF15^ and to RPGR fragments encoded by exons 1 through 15. No interaction was observed between INPP5E and the C-terminal region of RPGR (encoded by exon 16 to 19), suggesting that the N-terminal region of RPGR, likely the RCC1-like domain, contains the binding sites for INPP5E [15,20]. Interestingly, INPP5E knock-out cells were able to locate RPGR^ex1-19^ along the primary cilium, indicating that the ciliary localization of RPGR is not regulated by the binding partner INPP5E [15]. Our findings are in line with this hypothesis, because RPGR^skip14/15^ also located along the cilium and either did not or weakly interact with INPP5E. It seems more likely that RPGR influences the ciliary localization of INPP5E, as INPP5E was found to be decreased in concentration in dissociated mouse photoreceptors from *RPGR* knock-outs [20]. Nevertheless, studies in human RPGR knock-out cell lines found INPP5E along the primary cilium, indicating that the ciliary localization of INPP5E is not only regulated by RPGR [15]. It cannot be excluded that species- or cell-type-specific differences exist, but it seems more likely that RPGR and PDE6D co-regulate the localization of INPP5E. Interestingly, RPGR^skip14/15^ and INPP5E showed a loss of or reduced interaction, raising the possibility that a balanced expression of the different RPGR isoforms RPGR^ex1-19^ and RPGR^skip14/15^ fine-tunes the localization of INPP5E at the primary cilium.

Studies of protein networks suggested a functional relevance of RPGRIP1L for ciliary properties. RPGRIP1L is a known binding partner of RPGR and localizes to centrosomes of ciliated cells as well as to the basal body of primary cilia [38]. Apart from forming complexes with RPGR, RPGRIP1L interacts with NPHP1 and NPHP4, both of which are known to be crucial for ciliogenesis and normal cilia function [39,40,41], and with Myosin Va—an actin-based molecular motor which is also expressed at the centrosome [42]. The loss of function mutations in these proteins not only disrupts their interactions with RPGRIP1L, but was also associated with aberrant ciliogenesis and ciliopathies. RPGRIP1L has also been shown to be relevant for the formation and proper functioning of the ciliary transition zone in zebrafish development [43] and algae [44]. Further, interactions between RPGRIP1L and Psmd2 (which forms part of the proteasomal 19S subunit) have been implicated in the regulation of proteasomal activity at the base of the cilium [38]. Indeed, defects in the human *RPGRIP1L* gene were associated with the ciliopathies Joubert syndrome 7 and Meckel syndrome type 5 [39,40]. RPGRIP1L interacts with both RPGR^ex1-19^ and RPGR^ORF15^, isoforms that both include the RCC1-like domain [15]. In the present study, co-immunoprecipitations showed that RPGRIP1L is not an interaction partner of RPGR^skip14/15^, although RPGR^skip14/15^ contains the entire RCC1-like domain. Furthermore, the absence of RPGRIP1L in the interaction complex with RPGR^skip14/15^ does not prevent the localization of RPGR^skip14/15^ along the primary cilium. A knock-out of RPGRIP1L in RPE1 cells did not influence the ciliary localization of RPGR^ex1-19^ [15], but reduced the number of cells generating primary cilia [15]. Our cell lines uniquely express RPGR^skip14/15^ and showed no altered ciliogenesis. Again, the balanced expression of RPGR isoforms seems to regulate binding partners at the primary cilium, a notion that includes the binding partner RPGRIP1L.

Our observations lead to the hypothesis that RPGR isoforms differentially regulate binding partners at the primary cilium, which may influence functional properties of INPP5E and RPGRIP1L. RPGR might be the molecular link to fine-tune functions of the binding partners RPGRIP1L and INPP5E at the primary cilium. Different protein folding of RPGR^skip14/15^ and RPGR^ex1-19^ and/or differential accessibility of binding sites may be key to regulate the composition of binding complexes containing RPGR.

The data presented herein increase the understanding of the complex interplay between RPGR isoforms and their binding partners and have implications for the recent attempts to develop therapeutic approaches in order to help patients suffering from mutations in the *RPGR* gene.

## 4. Materials and Methods

### 4.1. Cell Culture and Transfection

Human embryonic kidney 293T cells (HEK293T) were cultured in Dulbecco’s modified Eagle medium high glucose (DMEM, Biowest, Nuaille, France) supplemented with 10% fetal bovine serum, 1.3% L-glutamine and 1.3% Pen/Strep and incubated at 37 °C and 5% CO_2_. To overexpress different *RPGR* transcripts in HEK293T cells, 1.0 × 10^6^ cells were seeded in a T25 flask and cultured for 24 h. HEK293T cells were transiently transfected using polyethyleneimine (PEI) (Sigma-Aldrich, St. Louis, MO, USA) with a DNA/PEI ratio of 1:4. After 6–8 h of incubation, the transfection medium was replaced with fresh media for 48 h. After 48 h, the cells were harvested for protein isolation.

### 4.2. Expression Constructs

To generate expression constructs encoding different RPGR transcripts, we performed PCRs to amplify the whole region of the human *RPGR* gene using Phusion Hot Start II DNA Polymerase (Thermo Fisher Scientific, Waltham, MA, USA). To delete exon 14 and 15 from the *RPGR* transcript, specific primers were designed. The first primer binds with its 3′-end to the first 25 bp of exon 16 and the 5′-end fits to the last 23 bp of exon 13 (5′-tatcaccagttcagaaacaaaaggatcatgaattttctaaaactgagg-3′). The second primer binds with its 3′-end to the last 23 bp of exon 13 and the 5′-end fits to the first 25 bp of exon 16 (5′-cctcagttttagaaaattcatgatccttttgtttctgaactggtgata-3′). The primers were either combined with the gateway cloning forward primer (5′-ggggacaagtttgtacaaaaaagcrggcttcatgagggagccggaagag-3′) or with the reverse primer (5′-ggggaccactttgtacaagaaagctgggtctagtattgtacaggattttgatcttc-3′). By overlapping-PCR, the deletion of exon 14 and 15 in the human *RPGR* sequence was generated using Phusion Hot Start II DNA Polymerase. Applying Gateway cloning strategies, the generated products were incorporated into the donor vector (pDONR221) to generate entry clones according to the manufacturer’s instruction (Gateway BP clonase II Enzyme mix; Invitrogen, Carlsbad, CA, USA). Using the Gateway LR Clonase Enzyme mix (Invitrogen, Carlsbad, CA, USA) sequence-verified entry clones were used to generate destination vectors (pcDNA3.1-FLAG-gate-pGK-HYG; #107397, Addgene) according to the manufacturer’s instruction.

### 4.3. Ciliary Staining and Microscopy

For immunocytochemical analyses of the primary cilium, Poly-L-lysine (PLL)-coated coverslips were placed at the bottom of a 12-well plate before cell seeding. 0.15 × 10^6^ HEK293T cells were seeded per well and cultured for 24 h. To induce cilia generation, the medium was replaced with nutrition-reduced culture medium. After 48 h, the cells were fixed for 20 min in 4% paraformaldehyde (PFA), washed three times in PBS-T (3 × 3 min with 1× Phosphate Buffered Saline (PBS) containing 0.05% Tween-20) and incubated for 30 min at 80 °C in 0.1 M Tris-HCL (pH 9). The cells were incubated for 30 min in PBS-T containing 2% bovine serum albumin (BSA) followed by an incubation with the primary antibodies at 4 °C overnight or at room temperature for 2 h. Cells were washed with PBS-T and the secondary antibodies conjugated either to Alexa 488 or Alexa 568 (Life Technologies, Carlsbad, CA, USA) were applied. Finally, the cells were mounted with Fluoromount containing 4′,6-Diamidin-2-phenylindol (DAPI) (SouthernBiotech, Birmingham, AL, USA). The axoneme of the primary cilium was stained using rabbit polyclonal anti-detyrosinated α-tubulin antibody (1:1000; Ab3201, Abcam, Cambridge, UK). RPGR was detected with the rabbit polyclonal RPGR antibody (1:400; HPA001593, Sigma-Aldrich, St. Louis, MO, USA). To measure the primary cilium frequency for each cell line, 150 cilia (50 cilia per coverslip from 3 coverslips) per experiment/replicate were counted in four independent experiments, in total 600 cilia per cell line. In order to achieve an unbiased counting of cilia, the number of cells was first determined from the DAPI channel, followed by a switch to the d-tubulin channel to check for the presence or absence of primary cilia on the cells. To analyze the localization of RPGR along the primary cilium, 50 cilia were counted for each cell line in four experiments, in total 200 cilia per cell line. Each experimental replicate was performed independently seeding the cells on separate days and at different passage numbers.

Images were obtained with a Zeiss Axio Observer 7 microscope (Zeiss, Oberkochen, Germany) using a 40×/0.6 Korr Ph2 M27 plan apochromat objective. Images were taken with the same exposure time and laser intensity. Representative positions on the cover slip were selected for the quantification, mostly in the middle of the cover slip. Identical cell density and antibody staining intensity was ensured across all experimental replicates. The edges of the coverslip, mostly with a thinner cell density, were omitted from the quantification. Images were processed using ZEN Blue software (version 2.3 SP1, Zeiss) and Fiji-ImageJ https://fiji.sc/, accessed on 15 January 2021). For a high degree of comparability, the contrast of the images was adjusted to a same level by the Brightness/Contrast-Tool of the Fiji software. The merged-channel tool of Fiji was used for overlaying the fluorescence images.

### 4.4. RPGR-Edited Cell Lines Using the CRISPR/eSpCas9 System

The expression vector eSpCas9(1.1) was purchased (#71814, Addgene, Watertown, MA, USA). To delete exon 14, 15 and ORF15 from the HEK293T cell genome, two sgRNAs were designed and cloned into the BbsI site of the eSpCas9(1.1)-plasmid: sgRNA 1 (5′-ATGTATCACAGACTAGAGAG-3′) and sgRNA 2 (5′-cagtacatttggttagttag-3′). The CRISPR/eSpCas9-mediated clones were generated as previously described [45]. HEK293T cells were transiently transfected using polyethyleneimine (PEI) (Sigma-Aldrich, St. Louis, MO, USA) with a DNA/PEI ratio of 1:4. The eSpCas9(1.1) plasmids (16.7 µg) containing either sgRNA 1 or sgRNA 2 were co-transfected with an eGFP-encoding plasmid (3.2 µg). After 24 h of cell culture, eGFP-positive cells were isolated by FACS (S3e cell sorter, BioRad, Hercules, CA, USA). After about 3 weeks of culturing the single cell sorted HEK293T cells, genomic DNA was isolated using the Puregene^®^ Blood Core Kit B (Qiagen, Hilden, Germany) according to the manufacturer’s instruction and used in PCR assays to amplify a fragment from intron 13 (5′-tggcaggtagtaagaatcgaaa-3′) to intron 15 (5′-ctagggaggccagtgttctc-3′) with HotFire Tag Polymerase (Solis Biodyne, Tartu, Estonia). To analyze potential off-target sites in the CRISPR/eSpCas9-mediated clones, PCR assays were performed using HotFire Tag Polymerase and analyzed on a 2% agarose gel. Afterwards the PCR products were verified by Sanger sequencing. Primer and PCR conditions used for the off-target analysis are listed in the Figure S1.

### 4.5. RNA Isolation and RT-PCR

Total RNA was extracted from cultured HEK293T cells using the NucleoSpin RNA kit (Macherey-Nagel, Düren, Germany) according to the manufacturer’s instruction. Using 500 ng total RNA in each sample, cDNA was reverse transcribed applying random primers and SuperScript III Reverse Transcriptase (Invitrogen, Carlsbad, CA, USA). To verify the CRISPR/Cas9-mediated clones, RT-PCRs were performed using HotFire Tag Polymerase (Solis Biodyne, Tartu, Estonia) to amplify two fragments from exon 5 (5′-tggtggaaataatgaaggacagt-3′) to exon 17 (5′-tggctttttctttcttttcaatagt-3′, primer bridging exons 16 and 17) representing either isoform RPGR^ex1-19^ or RPGR^skip14/15^. To verify whether the RPGR^skip14/15^ isoform is expressed in different human tissues, a multi-tissue RT-PCR was performed on pools of RNA from different human donor tissues (brain, kidney, lung, retina, testis; purchased from BioCat (Heidelberg, Germany)) using the following primer combination: (5′-acttgcttatctgtggcgacttttctg-3′) and (5′-tggctttttctttcttttcaatagt-3′). All PCR products were analyzed on a 2% agarose gel and sequence verified by Sanger sequencing. For quantitative RT-PCR, SYBR green was applied to detect both RPGR^skip14/15^ and GAPDH (internal control) on a CFX96 qPCR system (BioRad, Feldkirchen, Germany). Isoform RPGR^skip14/15^-specific amplification was achieved using a primer from exon 17 (5′-tggctttttctttcttttcaatagt-3′) in combination with a primer that binds with its 3′-end to exon 16 and with its 5′-end to exon 13 (5′-gttcagaaacaaaaggatcatg-3′).

### 4.6. Protein Isolation, Western Blotting and Co-Immunoprecipitation

For co-immunoprecipitation, FLAG-tagged expression constructs encoding RPGR^ex1-19^ or RPGR^skip14/15^ were transfected into HEK293T cells using a DNA/PEI ratio of 1:4. For each co-precipitation, 400 µg clear protein lysate was incubated with anti-FLAG Beads (Bimake, Houston, TX, USA) for 2 h at 4 °C. The beads were washed three times with SLB and eluted for 5 min at 95 °C. To generate protein lysates, cells were lysed in Spheroid Lysis Buffer (SLB) buffer (40 mM HEPES, 120 mM NaCl, 0.3% CHAPS, pH 7.5) including phosphatase inhibitor cocktails 2 and 3 (1:100; Sigma-Aldrich, St. Louis, MO, USA) and Complete Protease inhibitor cocktail (1:25; Roche, Basel, Switzerland). The lysates were incubated for 20 min on ice and centrifuged at 600 g for 3 min. The protein concentration was measured using a bicinchoninic acid assay(BCA) protein assay kit (Thermo Fisher Scientific, Waltham, MA, USA) according to the manufacturer’s instruction. Protein lysates were mixed with 5× SDS buffer, incubated for 5 min at 95 °C and loaded for separation on a 10% SDS-polyacrylamide gel. The proteins were transferred on a Polyvinylidene difluoride (PVDF)-membrane (0.45 µm pore size; Millipore, Burlington, VT, USA) by wet blotting at 45 V for 2 h. The membrane was blocked in 5% bovine serum albumin in TBST for 30 min and incubated with the primary antibodies overnight at 4 °C. A horseradish peroxidase-conjugated secondary antibody was used for detection by enhanced chemiluminescence (ECL) reaction. Reactive bands were visualized with the ChemiDoc MP chemiluminescence detection system (BioRad Laboratories, Munich, Germany).

RPGR^ex1-19^ and RPGR^skip14/15^ were detected by a rabbit polyclonal RPGR antibody (1:500; HPA001593, Sigma-Aldrich, St. Louis, MO, USA). Additional primary antibodies were: rabbit polyclonal RPGRIP1L antibody (1:500; 55160-1-ap, proteintech, Rosemont, IL, USA), rabbit polyclonal PDE6D antibody (1:1000; MBS7005086, Mybiosource, San Diego, CA, USA), rabbit polyclonal INPP5E antibody (1:500; 17797-1-ap, proteintech, Rosemont, IL, USA) and mouse monoclonal GAPDH antibody (1:1000; 0508007983, Chemicon International, Temecula, CA, USA).

For Western blotting (immunoblotting), cells were lysed in radio immunoprecipitation assay (RIPA) buffer (1% NP40, 0.1% sodium dodecyl sulphate (SDS), 0.5% sodium deoxychalate in PBS) supplemented with protease inhibitor cocktail and phosphatase inhibitor cocktails 2 and 3 (as described in the Co-IP assay above). The resultant lysates were incubated on ice for 5 min and centrifuged at 6000× *g* for 10 min. The supernatant was collected and the protein concentration measured using Quick Start Bradford 1× Dye Reagent (Bio-Rad Laboratories, Munich, Germany). The protein concentration was adjusted to the sample with the lowest value. The lysates were then mixed with sample buffer (10% glycerol, 1% beta-mercaptoethanol, 1.7% SDS, 6.25% 1 M Tris (pH 6.8), 3.34% bromophenol blue) and heated for 5 min at 95 °C. Samples were then loaded onto 10% SDS-polyacrylamide gels and separated at 90 V for 1 h, followed by 180 V for 30 min. Protein transfer, membrane blocking, primary antibody and secondary antibody incubations were performed as described above in the Co-IP assay. Protein bands were detected using INTAS ECL CHEMOCAM IMAGER (INTAS Science Imaging Instruments, Göttingen, Germany).

### 4.7. Statistical Analysis

For the quantification of the subcellular localization of RPGR and the cilium frequency, at least four independent experimental replicates were performed. Random pictures were taken, and 50 cells were analyzed per experiment. The significance of quantitative RT-PCRs was calculated using a *t*-test. Quantification of Western blot band intensities was performed using either the LabImage 1D software (INTAS Science Imaging Instruments, Göttingen, Germany) or the Image Lab software (Bio-Rad Laboratories, Munich, Germany) depending on which imager was used for acquiring the immunoblot images. The quantification software tools were used to measure the band volumes of the respective protein bands with background signal correction applied. The resultant values were then normalized to their respective loading controls. Statistical significance was calculated using Prism software (Graphpad Software, San Diego, CA, USA) applying ordinary one-way ANOVA using Tukey‘s multiple comparisons test or unpaired *t*-test with Welch’s correction. *p*-values of <0.05 were considered statistically significant.

## Data Availability

The data presented in this study are available on request from the corresponding author.

## Supplementary Materials

Supplementary materials can be found at https://www.mdpi.com/article/10.3390/ijms22073583/s1.

## Abbreviations

X-linked Retinitis pigmentosa (XLRP); retinal degenerative disorders (RD); retinitis pigmentosa GTPase regulator (RPGR); RPGR including exon 1 through 19 (RPGR^ex1-19^); RPGR including exon 1 through ORF15 (RPGR^ORF15^); RPGR including exon 1 through 19, but excluding exons 14 and 15 (RPGR^skip14/15^); clustered regularly interspaced short palindromic repeats/CRISPR-associated protein 9 (CRISPR/Cas9); HEK 293 cell line: that expresses a mutant version of the SV40 large T antigen (HEK293T); Retinal rod rhodopsin-sensitive cGMP 3′,5′-cyclic phosphodiesterase subunit delta (PDE6D); Inositol polyphosphate-5-phosphatase E (INPP5E); RPGR-Interacting Protein 1-Like Protein (RPGRIP1L); Regulator Of Chromosome Condensation 1-like domain (RLD); guanine nucleotide exchange factor (GEF); Polymerase chain reaction (PCR); Reverse transcriptase PCR (RT-PCR); single-guide RNAs (sgRNAs); intraflagellar transport (IFT); green fluorescence protein (GFP); Dulbecco’s modified Eagle medium high glucose (DMEM); polyethyleneimine (PEI); Poly-L-lysine (PLL); paraformaldehyde (PFA); phosphate buffered saline (PBS); fluorescence-activated cell sorting (FACS); 4′,6-Diamidin-2-phenylindol (DAPI).
