# Supplementary material for: The Major Ciliary Isoforms of RPGR Build Different Interaction Complexes with INPP5E and RPGRIP1L
Source: Int J Mol Sci. 2021 Mar 30;22(7):3583. doi: 10.3390/ijms22073583 (PMC8037643; doi:10.3390/ijms22073583)
Supplement: Supplementary file 1 [file ijms-22-03583-s001.pdf]

Supplementary material

The major ciliary isoforms of RPGR build different interaction complexes with INPP5E and RPRGIP1L

C. Vössing[1], P. Atigbire[1], J. Eilers[1], F. Markus[2], K. Stieger[3], F. Song[1], J. Neidhardt[1,4,5 #]

[1] Human Genetics, Faculty VI-School of Medicine and Health Sciences, University of Oldenburg, 26129 Oldenburg, Germany

[2] Junior Research Group, Genetics of Childhood Brain Malformations, Faculty VI-School of Medicine and Health Sciences, University of Oldenburg, 26129 Oldenburg, Germany

[3] Department of Ophthalmology, Justus-Liebig-University Giessen, 35390 Giessen, Germany

[4] Research Center Neurosensory Science, University of Oldenburg, 26129 Oldenburg, Germany

[5] Joint Research Training Group of the Faculty of Medicine and Health Sciences, University of Oldenburg, Germany and the University Medical Center Groningen, Groningen, Netherlands

*	Correspondence: john.neidhardt@uni-oldenburg.de, Tel.: +49 (0)441-798-3800

sgRNA: RPGR-Intron 13

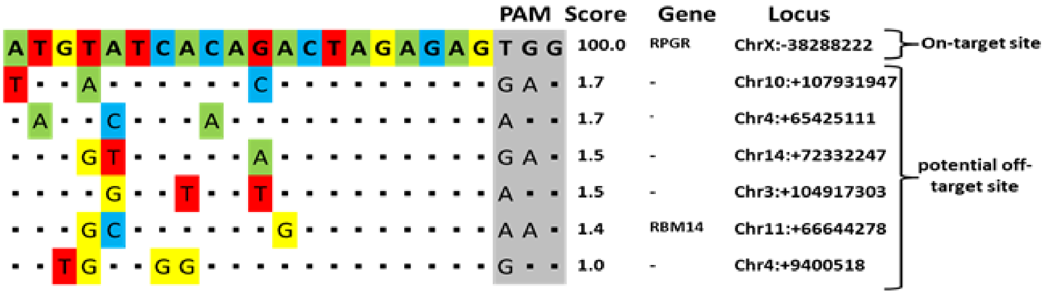

Locus	Forward Primer 5'-3'	Reverse Primer 5'-3'	Comment	Size (bp)
Chr10:+107931947	ttctggtgtgtgtgttggca	tgtgaggcagggagtcatta	65°C annealing	357
Chr4:+65425111	tcacgcatccctatagccaac	gcatagcagtgagaagtggg	65°C annealing	393
Chr14:+72332247	gctagacactgttgagccca	tccatctcactgctttccct	65°C annealing	364
Chr3:+104917303	ccaacaacagagccataaccc	agtgagagaagagaatggcct	60°C annealing	340
Chr11:+66644278	gcgcggaattctctgtacga	acccaagattcccagcaca	66°C annealing	366
Chr4:+9400518	cagagcatctgaaaccttgtaag	ctaggaatattaccaggaaggag	60°C annealing	434

sgRNA: RPGR-Intron 15

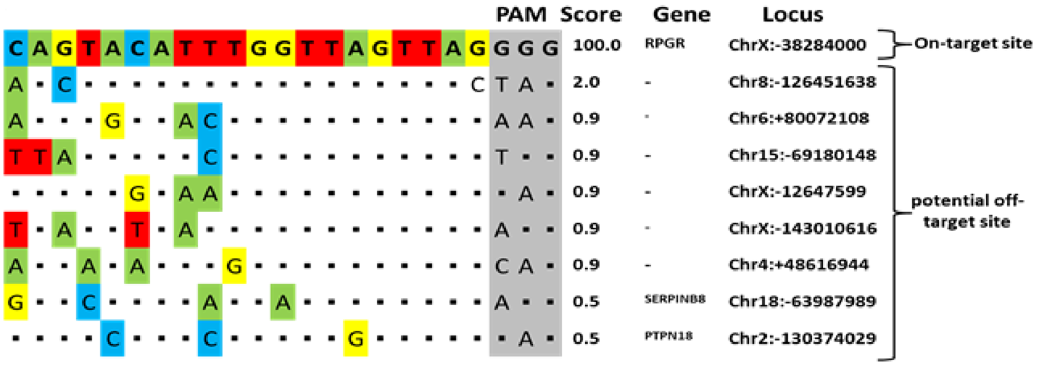

Locus	Forward Primer 5'-3'	Reverse Primer 5'-3'	Comment	Size (bp)
Chr8:-126451638	aagcaggaacagggttgaca	acagacagctaggcctctct	66°C annealing	304
Chr6:+80072108	tgctgctgtctatgctggtt	tgggtaagcttgtgattcagt	65°C annealing	381
Chr15:-69180148	ggaggctgaggcacaagaattgc	gaggtacttacagagcctagagc	68°C annealing	449
ChrX:-12647599	acaccagccagagactagaga	gagaagccaggagaatgcca	67°C annealing	534
ChrX:-143010616	agcaacagcaagagagggtc	gagtcatcaaggcaagctgg	65°C annealing	363
Chr4:+48616944	tcctctgtagactgtgtgcct	aatacagtggccttgatagctaa	66°C annealing	475
Chr18:-63987989	tccataagcctgagatacaagtt	tgctgtgtgttggaattgtgg	59°C annealing	334
Chr2:-130374029	tagaccagtgcagccagaga	tgtctgaatagctggagatgtgt	68°C annealing	390

Supplementary Figure S1: Scores of potential off-target sites were calculated using the Benchling software (https://www.benchling.com/) based on the algorithms developed by Doench *et al*. and Hsu *et al*. (Doench et al., 2014; Hsu et al., 2013). The off-target score is between 0 to 100 and represents the probability of the Cas9 binding to induce double strand brakes. We considered potential off-target sites for sgRNA1 (RPGR-Intron13) and sgRNA2 (RPGR-Intron15) with up to 4 bp mismatches.

Mismatches are indicated in the coloured boxes (green, blue, red, yellow). The PAM sequence is highlighted in grey. The genomic locus of potential off-targets in the human genome is listed (Reference sequence: GRCh38 (hg38, Homo sapiens). In the table, primer combinations for all potential off-target sites are listed. Sequence alternation at the potential off-target sites were not detected.

References

Doench, J. G., Hartenian, E., Graham, D. B., Tothova, Z., Hegde, M., Smith, I., . . . Root, D. E. (2014). Rational design of highly active sgRNAs for CRISPR-Cas9-mediated gene inactivation. *Nat Biotechnol, 32*(12), 1262-1267. doi:10.1038/nbt.3026

Hsu, P. D., Scott, D. A., Weinstein, J. A., Ran, F. A., Konermann, S., Agarwala, V., . . . Zhang, F. (2013). DNA targeting specificity of RNA-guided Cas9 nucleases. *Nat Biotechnol, 31*(9), 827-832. doi:10.1038/nbt.2647

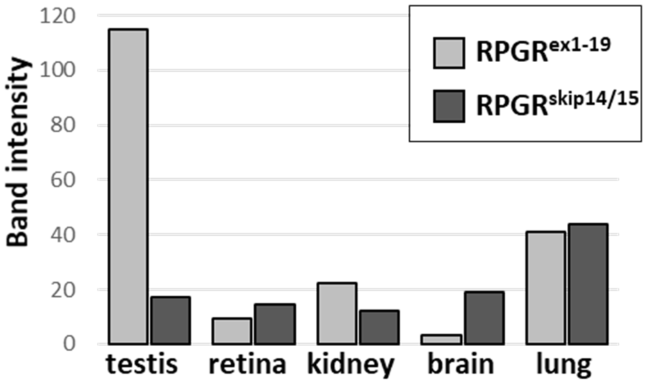

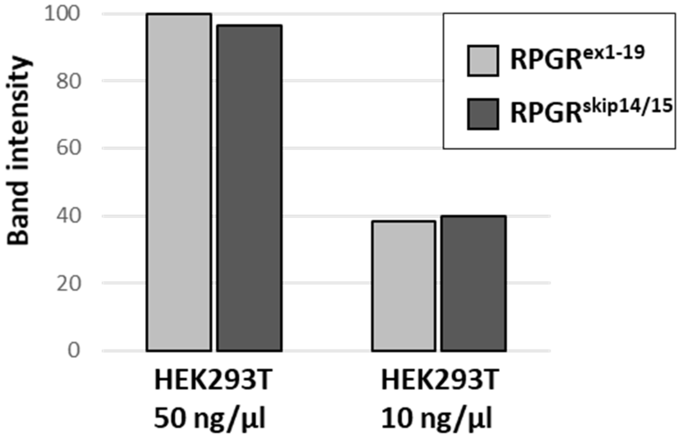

Supplementary Figure S2: Densitometric measurement of RT-PCR product intensities of *RPGR*$^{skip14/15}$ and *RPGR*$^{ex1\text{-}19}$ isoforms from different tissues and HEK293T cells. For RT-PCR results, please refer to figure 1. The upper panel shows the comparison of band intensities (arbitrary units) found in RT-PCR analyses of *RPGR*$^{skip14/15}$ and *RPGR*$^{ex1\text{-}19}$ in different human tissues. The lower panel show the comparison between *RPGR*$^{skip14/15}$ and *RPGR*$^{ex1\text{-}19}$ band intensities for HEK293T cells, where we used two different cDNA concentrations per reaction (10 ng and 50 ng).

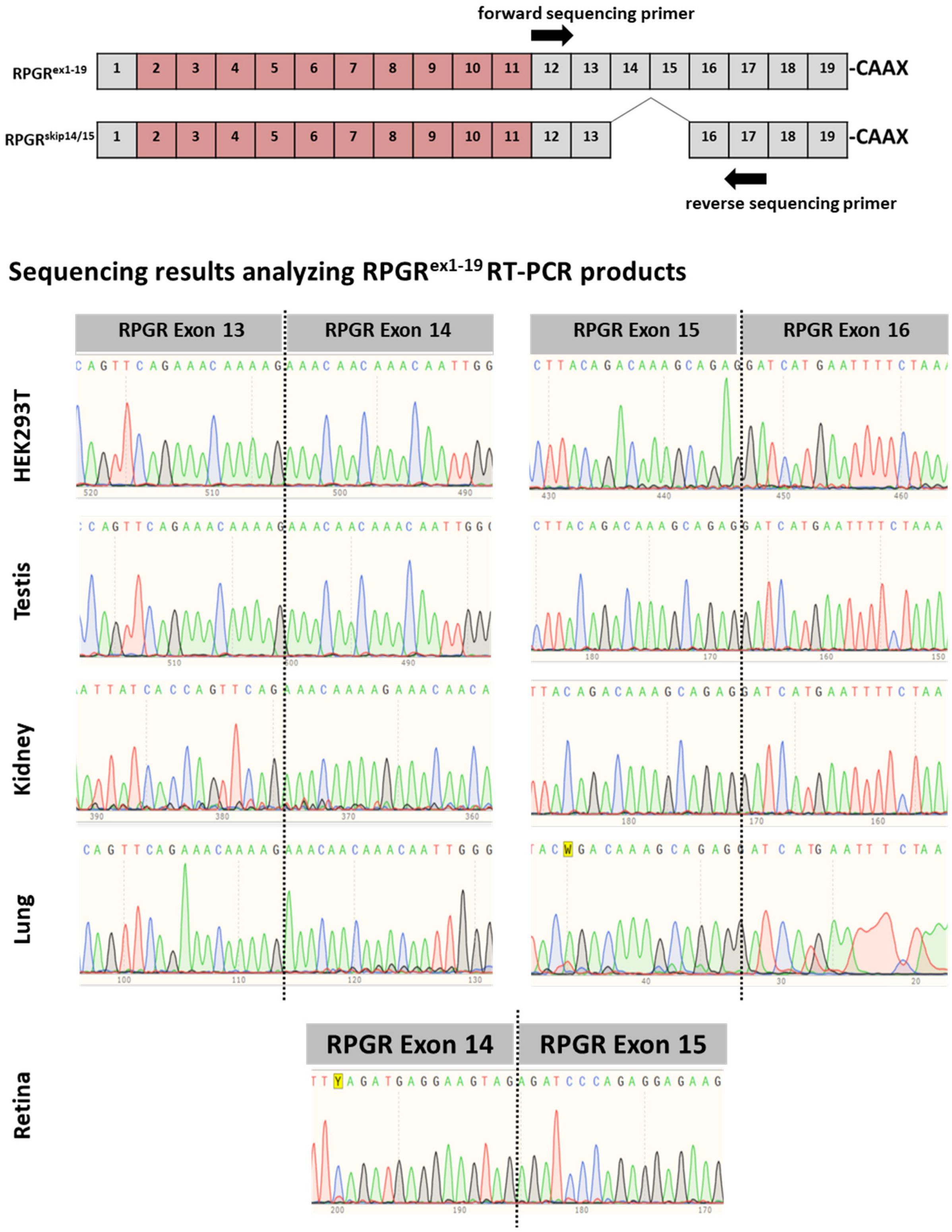

Supplementary Figure S3: Sanger sequencing results of RT-PCR products of the *RPGR*$^{ex1\text{-}19}$ isoform from different human tissues and HEK293T cells. For RT-PCR results, please refer to figure 1. The sequence profiles (electropherograms) confirmed the identity of the *RPGR*$^{ex1\text{-}19}$ splice product detected in different human tissues and HEK293T cells.

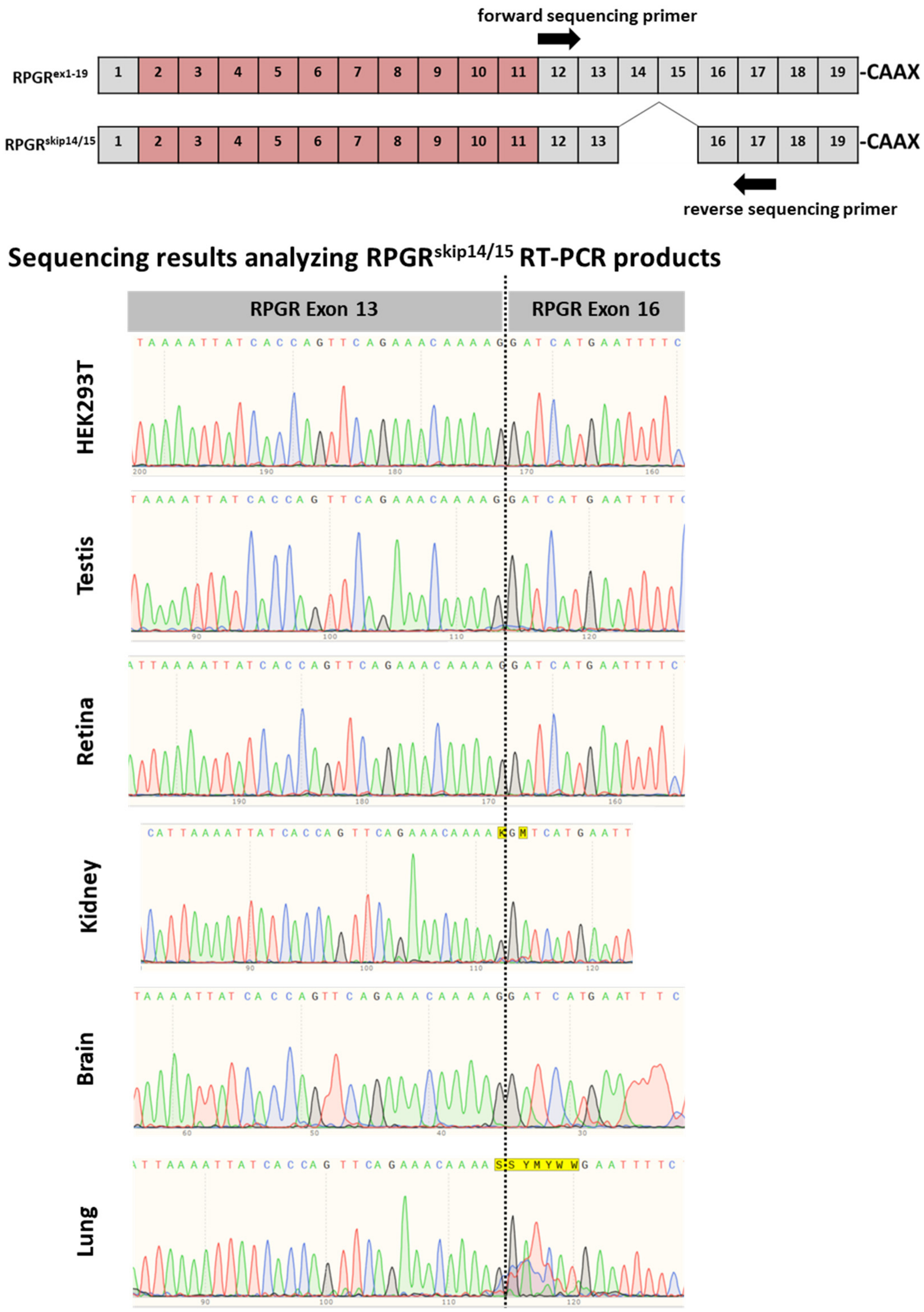

Supplementary Figure S4: Sanger sequencing results of RT-PCR products of the *RPGR*$^{skip14/15}$ isoform from different human tissues and HEK293T cells. For RT-PCR results, please refer to Figure 1. The sequence profiles (electropherograms) confirmed the identity of the *RPGR*$^{skip14/15}$ splice product detected in different human tissues and HEK293T cells.

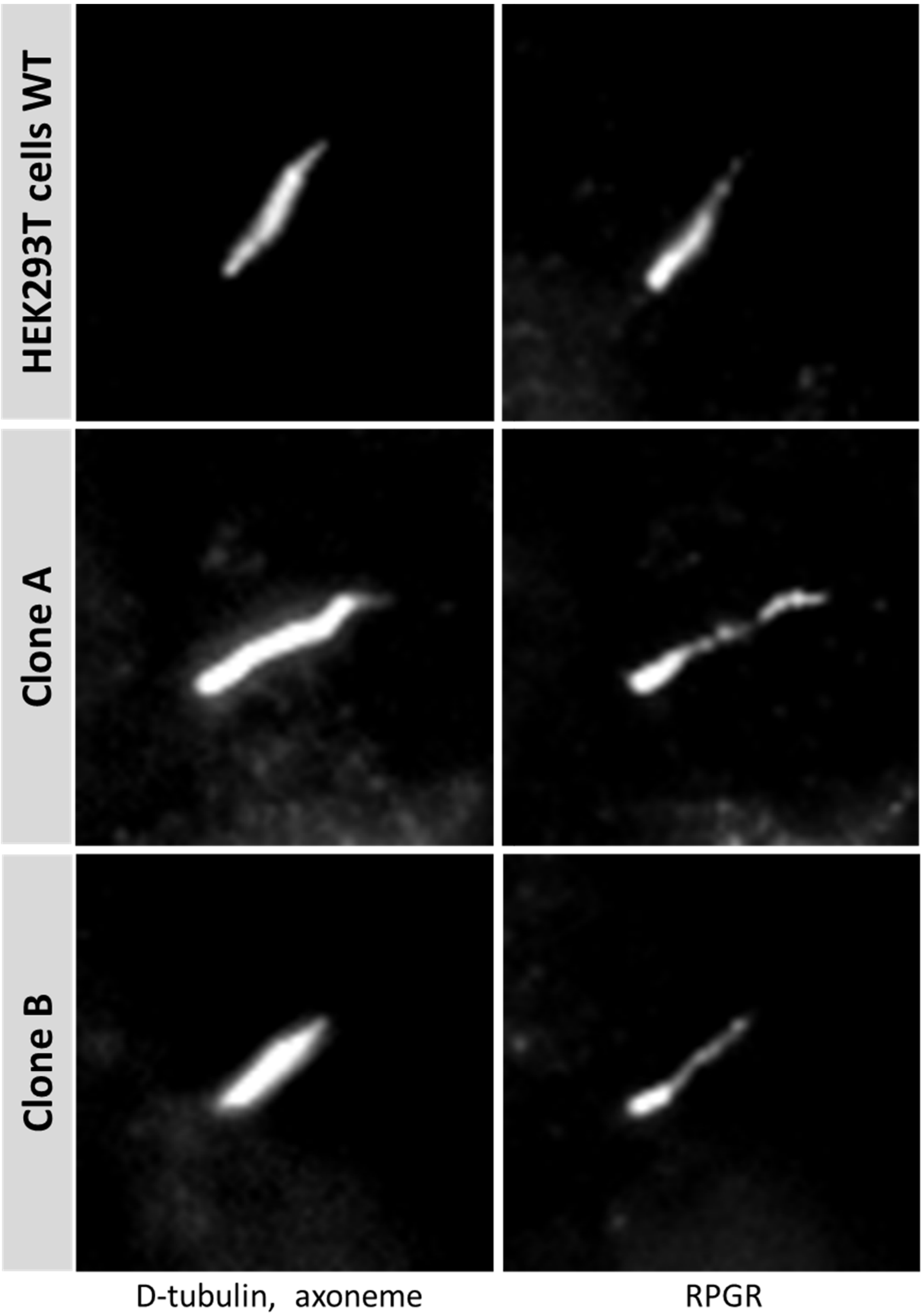

Supplementary Figure S5: Higher magnification micrographs of immunocytochemical signals detecting RPGR and the axoneme (d-tubulin) in unaltered HEK293T cells, in clone A, and in clone B. Similar results were detected between the different cell lines suggesting that the ciliary localization of RPGR was not disturbed by the CRISPR/eSpCas9-induced genomic alterations of clones A and B.
